# Selective intra-arterial hypothermia combined with endovascular thrombectomy for large vessel occlusion: A systematic review and meta-analysis

**DOI:** 10.1177/15910199241285157

**Published:** 2024-09-19

**Authors:** Fahad Alturki, Ahmed Alkhiri, Bander Alsulami, Fawaz F. Alotaibi, Aser F. Alamri, Bader AlRuhaymi, Elyas M. Bakhuraybah, Fahad S. Al-Ajlan, Adel Alhazzani, Mohammed A. Almekhlafi

**Affiliations:** 1 48150 College of Medicine, King Saud Bin Abdulaziz University for Health Sciences, Riyadh, Saudi Arabia; 2 309817 King Abdullah International Medical Research Center, Riyadh, Saudi Arabia; 3 48150 College of Medicine, King Saud Bin Abdulaziz University for Health Sciences, Jeddah, Saudi Arabia; 4 309817 King Abdullah International Medical Research Center, Jeddah, Saudi Arabia; 5 Neuroscience Center, 37852 King Faisal Specialist Hospital and Research Centre, Riyadh, Saudi Arabia; 6 Alfaisal University, Riyadh, Saudi Arabia; 7 Department of Clinical Neurosciences, University of Calgary, Calgary, AB, Canada

**Keywords:** Stroke, large vessel occlusion, endovascular thrombectomy, hypothermia, neuroprotection, selective hypothermia

## Abstract

**Background:**

Systemic therapeutic hypothermia may improve outcomes after acute ischemic stroke but increases complications. Selective intra-arterial hypothermia at the ischemic site during endovascular thrombectomy (EVT) theoretically offers benefits with fewer risks. However, there is little clinical evidence to support this approach.

**Methods:**

We searched Medline/PubMed, Embase and Cochrane electronic databases for studies evaluating the safety and feasibility of selective intra-arterial hypothermia as an adjunct to EVT for large vessel occlusion (LVO). Effect sizes with 95% confidence intervals (CIs) were pooled using the fixed-effect model. Odds ratios (ORs) were computed for binary variables, while the mean differences (MDs) were pooled for continuous data.

**Results:**

Of identified records, five clinical studies involving 463 LVO patients (62.9% male) were included. Of those, 224 (48.4%) patients received adjuvant selective intra-arterial hypothermia, while 239 (51.6%) received EVT alone. Selective intra-arterial hypothermia resulted in higher rates of good functional outcome (modified Rankin scale [mRS] 0–2 at 90-days) (OR 2.07, [95% CI, 1.36 to 3.16]), and lower final infarct volume (MD, −20.96 ml [95% CI, −26.17 to −15.75]) and lower rates of severe disability (mRS 3–5 at 90 days) (OR 0.44 [95% CI, 0.26 to 0.75]). Safety parameters including rates of symptomatic intracerebral hemorrhage, mortality, pneumonia, coagulation abnormalities, and arterial spasm were comparable between groups.

**Conclusions:**

The initial evidence supports the safety and feasibility of selective intra-arterial hypothermia when combined with EVT for LVO. This approach shows promise for advancing research on neuroprotective strategies for ischemic stroke.

## Introduction

Based on multiple randomized clinical trials (RCTs), endovascular thrombectomy (EVT) is recognized as the standard of care for acute ischemic stroke (AIS) caused by large vessel occlusions (LVOs).^1–5^ Recently, the role of EVT has expanded to include patients with basilar artery occlusion^6^ and large ischemic core infarcts.^7^ However, long-term prognosis remains challenging for LVO patients, as less than half of patients who undergo EVT achieve good functional outcomes, and mortality rates reach 15%.^8^ This takes on particular relevance for patients with basilar occlusion^9,10^ or large infarcts,^11,12^ as a substantial portion experience severe disability with high mortality rates despite EVT treatment. Accordingly, there remains hope for adjunctive neuroprotective strategies which may help optimize outcome.

In the 1980s, Busto and colleagues found that reducing brain temperature by only a few degrees during ischemia may have neuroprotective effects.^13^ Therefore, interest grew in translating this approach to stroke patients. Experimental research suggests that therapeutic hypothermia can affect various metabolic and molecular pathways implicated in ischemic neuronal injury. However, clinical studies to date have not conclusively demonstrated a net benefit for hypothermia in AIS patients as seen in other populations such as post-cardiac arrest, neonatal hypoxic-ischemic encephalopathy patients, and traumatic brain injury.^14–16^ Prior attempts with systemic hypothermia for AIS patients were limited due to the high rates of complications which offset any benefits.^17,18^ Selective intra-arterial hypothermia at the ischemic site during EVT theoretically offers the benefits of hypothermia with fewer risks than systemic cooling.^19–23^ However, there is little clinical evidence to support this approach. In this systematic review and meta-analysis, we aimed to evaluate the safety and feasibility of selective intra-arterial hypothermia combined with EVT in patients with LVO-AIS.

## Methods

This systematic review and meta-analysis adhered to the Preferred Reporting Items for Systematic Reviews and Meta-Analyses (PRISMA) and the Meta-analysis of Observational Studies in Epidemiology (MOOSE) reporting guidelines.^24,25^ The protocol was registered with PROSPERO (CRD42024549788). As this work constitute a systematic review and meta-analysis of previously published studies, ethical approvals were not required, and data are available within the manuscript and included articles.

### Data sources and searches

Medline, Embase, and Cochrane Library were searched for English articles from inception until April 2024. We used combination of terms related to Stroke, Endovascular thrombectomy, and Selective Hypothermia to obtain articles from each database. Detailed search strategy can be found in the Supplemental material. To identify additional relevant studies that were not captured in the initial database searches, we performed a bibliography screening and manually searched Google Scholar.

### Study selection and eligibility

After eliminating duplicates, two independent authors screened titles and abstracts. Potentially eligible records were retrieved for full-text assessment. We included studies that reported outcomes of LVO-AIS patients treated with EVT and selective intra-arterial hypothermia. We excluded studies involving systemic therapeutic hypothermia, surface cooling and any other hypothermia approach that does not involve intra-arterial infusion, treatment with intravenous thrombolysis alone, non-human studies, conference abstracts, review articles and studies not published in English.

### Data extraction and risk of bias

Data on study and patients’ characteristics were independently extracted by two authors using a standardized form. The quality of the included RCTs was assessed using the Cochrane Risk of Bias 2 (RoB2) tool.^26^ The RoB2 tool evaluates five bias domains and categorizes the overall risk of bias as ‘Low risk’ ‘Some concerns’ or ‘High risk’ based on assessments in each domain. The quality of the included observational studies was assessed using the Newcastle-Ottawa scale (NOS).^27^ The NOS adopts a star system from 0 to 9, where scores of 7 stars or more indicate good quality. Any discrepancies were resolved through consensus with a third author.

### Statistical analysis and outcomes

Statistical analysis was performed using the RevMan 5.4 software. Study outcomes included good functional outcome (modified Rankin Scale [mRS] of 0–2 at 90 days), final infarct volume, severe disability (mRS of 3–5 at 90 days), all-cause mortality, symptomatic intracerebral hemorrhage (sICH), pneumonia, urinary tract infections, arterial vasospasm, and abnormal coagulation parameters. Preferably, adjusted values were synthesized instead of unadjusted values. Effect estimates were presented as odds ratios (ORs) with corresponding 95% confidence intervals (CIs) for binary variables, while continuous data were described as mean differences (MDs) with 95% CIs. Heterogeneity was evaluated using I^2^ statistics and the p-value of χ^2^ test. Pooled effects were considered homogonous, and thus the fixed effects model was employed when I^2 ^< 50% and p > 0.1. Whereas an I^2 ^≥ 50% or p ≤ 0.1 implied that the pooled result had heterogeneity, and therefore, the random-effect model was used.

## Results

### Study selection

**Figure 1.**
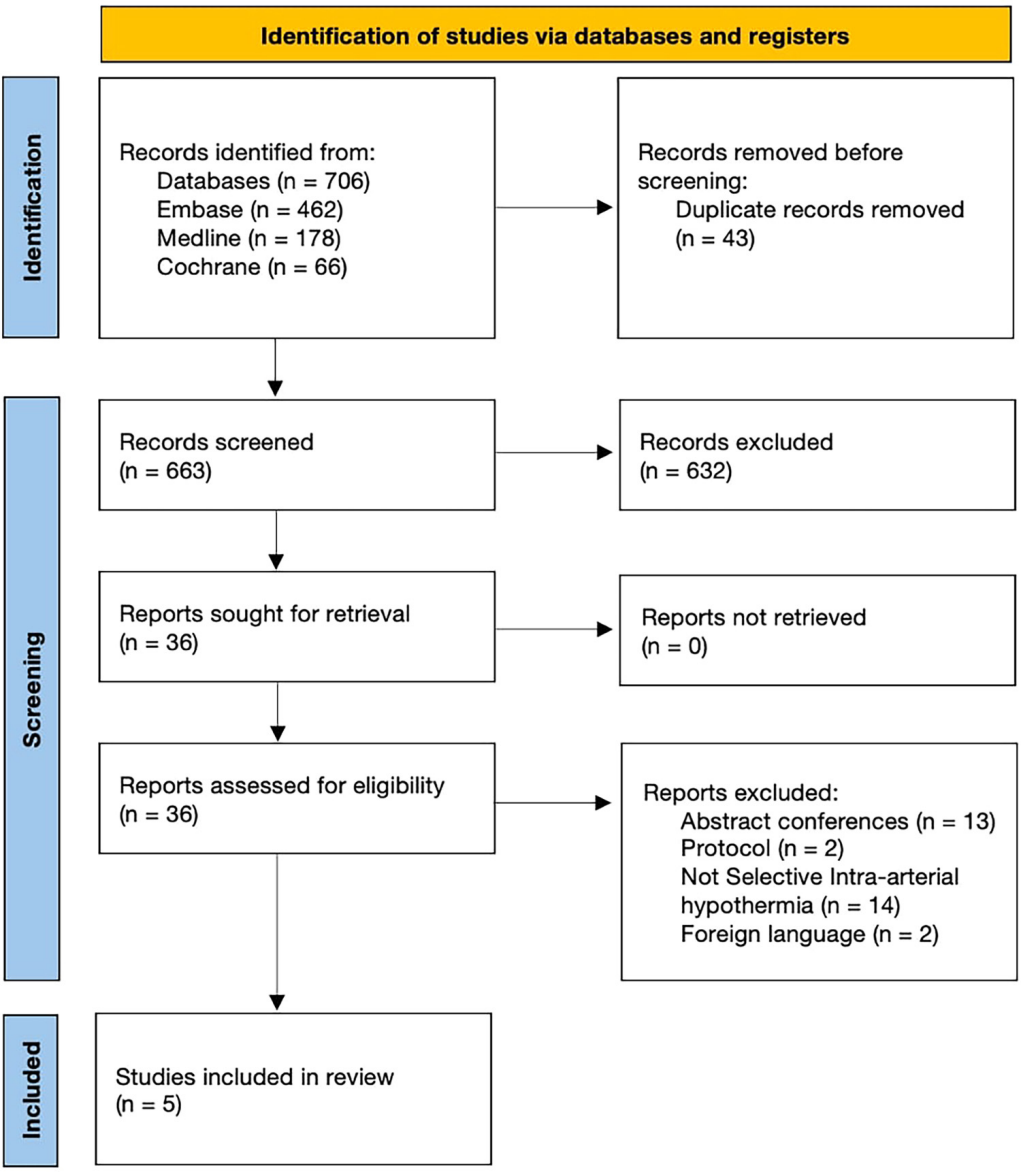
Prisma flow diagram of study selection process.

The database search identified a total of 706 studies. After removing duplicate records, 663 titles and abstracts were screened for eligibility. Of these, 36 full-text articles underwent full-text assessment for eligibility. Subsequently, 5 studies^19–23^ satisfied our inclusion criteria and were included in the final analysis (Figure 1).

### Study characteristics and risk of bias

A summary of the included studies is provided in Table 1. One RCT^19^ and four observational studies^20–23^ published between 2016 and 2023 with a total of 463 LVO patients were included. Of these, 224 (48.4%) patients received adjuvant selective intra-arterial hypothermia with EVT, while 239 (51.6%) treated with EVT alone. There were 293 (62.9%) male participants. The mean age of included patients ranged from 56.1 to 73.4 years. All included studies were conducted in China. Two studies^19,22^ originated from the same center, however there was no significant patient overlap between the two samples as indicated by the authors. Time to recanalization was higher in patients treated with selective intra-arterial hypothermia. Detailed baseline characteristics including age, stroke severity, and comorbidities were generally balanced between both groups as shown in Table S4.

**Table 1.** Overview of included studies.

Study ID	Country	Study Duration	Study design	Patients N (intervention/control)	Bridging Intravenous thrombolysis, N (%)	ASPECTS, Median (Range)	Main inclusion criteria
Wu et al., 2018	China	2015–2017	Single-center Prospective Study	45/68	50 (44.2)	Intervention 8 (7–10) Control: 9 (8–10)	1) LVO within 6 h from onset 2) NIHSS ≥6 3) Occlusion in M1
Wan et al., 2023	China	2019–2022	Single-center Randomized Trial	71/71	0 (0)	NR	1) LVO within 24 h from onset 2) NIHSS ≥6 3) Occlusion in M1, M2, ICA
Chen et al., 2016	China	2013–2014	Single-center Retrospective Study	26/-	NR	Intervention: 6 (6–9)	1) LVO within 8 h from onset 2) NIHSS ≥8 3) Occlusion in M1, M2, ICA, BA, VA
Li et al., 2023	China	2020–2022	Single-center Single-arm Prospective Study	20/20	NR	Intervention 8.4 (1.05) * Control: 8.4 (0.83) *	1) LVO achieving TICI 2b
Tian et al., 2023	China	2018–2022	Single-center Prospective Study	62/80	29 (20.4)	NR	1) Within 24 h from onset 2) NIHSS ≥6 3) Occlusion in M1

NR indicates Not reported; ASPECTS, Alberta stroke program early computerized tomography score; LVO, Large vessel occlusion; NIHSS, National institutes of health stroke scale; M1, First segment of the middle cererbral artery; M2, Second segment of the middle cererbral artery; ICA, Internal caratoid artery; BA, Basilar artery; VA, Vertebral artery; TICI, Thrombolysis in cerebral infarction.

* Data is described as mean (standard deviation).

The risk of bias assessment can be found in the Supplement. In short, the RCT^19^ was graded as having ‘some concern’ due to potential bias in the randomization and selection domains. All observational studies,^21–23^ except for the study by Chen et al.,^20^ met the criteria for good quality.

### Hypothermia protocol

Table 2 presents detailed treatment protocols. All included studies applied chilled saline at 4°C.^19–23^ Two studies utilized an interrupted hypothermia protocol,^19,22^ involving the infusion of 4°C normal saline via a catheter before EVT and after successful recanalization. Another two studies employed a continuous hypothermia protocol,^20,23^ where 4°C saline infusion through the microcatheter was maintained continuously before EVT and via the catheter after recanalization. One study^21^ solely implemented continuous cooling after recanalization through the guide catheter.

**Table 2.** Detailed description of selective intra-arterial hypothermia treatment across included studies.

Study ID	Hypothermia method	Total infusion duration	Total infusion volume	Description of intra-arterial infusion	Time metrics, min
Wu et al., 2018	Continuous infusion (pre and post-EVT)	15 min	350 ml	4°C cold saline was infused at 10 ml/min pre-EVT and reinfused at 30 ml/min for 10 min after successful recanalization (mTICI 3).	From stroke onset to hypothermia: 341 (253–391)^§^
Wan et al., 2023	Interrupted infusion (pre and post-EVT)	35 min	515 ml	4°C cold saline was infused at 15 ml/ min for 5 min pre-EVT and reinfused at 22 ml/min for 20 min with 10 min interval between each 10 min after recanalization.^#^	From stroke onset to groin puncture: 258 (71)*
Chen et al., 2016	Continuous infusion (pre and post-EVT)	15 min	350 ml	4°C cold saline was infused at 10 ml/min pre-EVT and reinfused at 30 ml/min for 10 min after successful recanalization (TICI 2b).	From stroke onset to groin puncture: 383 (195–432)§
Li et al., 2023	Continuous infusion (pre and post-EVT)	20 min	500 ml	4°C cold saline was infused at 25 ml/min after successful recanalization (TICI 2b).	From stroke onset to hypothermia: 450 (244.2)*
Tian et al., 2023	Interrupted infusion (pre and post-EVT)	20 min	500 ml	4°C cold saline was perfused at 10 ml/min for 5 min pre-EVT, after successful recanalization (mTICI 2b-3) it was reinfused at 30 ml/min three times for 5 min with 10 min intervals.	From stroke onset to hypothermia: 266 (208–295)^§^

EVT indicates Endovascular thrombectomy; NR, Not reported; (m)TICI, (Modified) thrombolysis in cerebral infarction.

* Data is described as mean (standard deviation).

§ Data described as median (range).

# Did not provide a definition for successful recanalization.

### Study outcomes

**Figure 2.**
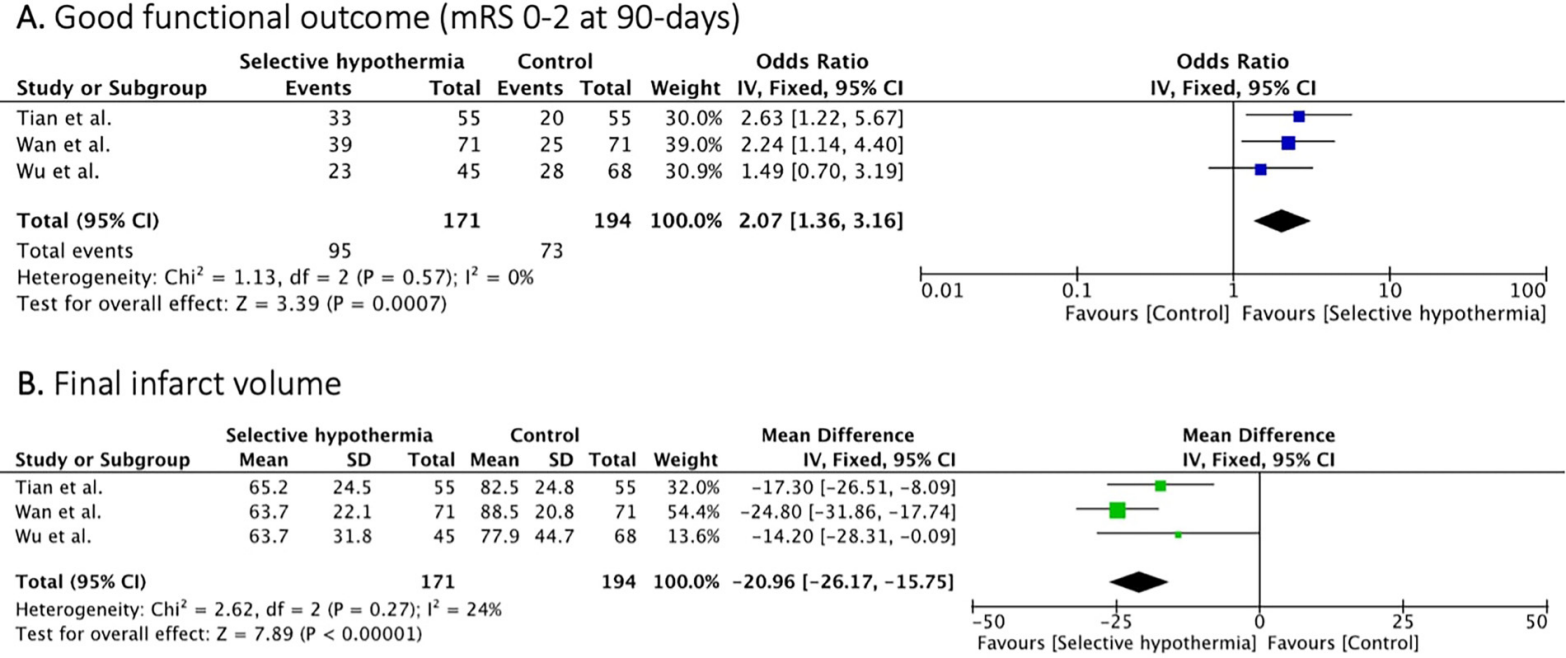
Forest plot of rates of good functional outcome (mRS 0-2) and final infarct volume.

Three studies^19,22,23^ (365 patients) reported on the good functional outcome (mRS 0–2 at 90 days). Selective intra-arterial hypothermia resulted in higher rates of good functional outcome compared to EVT treatment alone (OR 2.07, [95% CI, 1.36 to 3.16]; p = 0.0007), with no heterogeneity (I^2 ^= 0%, p = 0.57) (Figure 2A).

Three studies^19,22,23^ (365 patients) reported on the final infarct volume. Patients treated with selective intra-arterial had lower mean final infarct volume compared to patients treated with EVT alone (MD −20.96 ml [95% CI, −26.17 to −15.75]; p = 0.00001) with no significant heterogeneity observed between studies (I^2 ^= 24%, p = 0.27) (Figure 2B).

**Figure 3.**
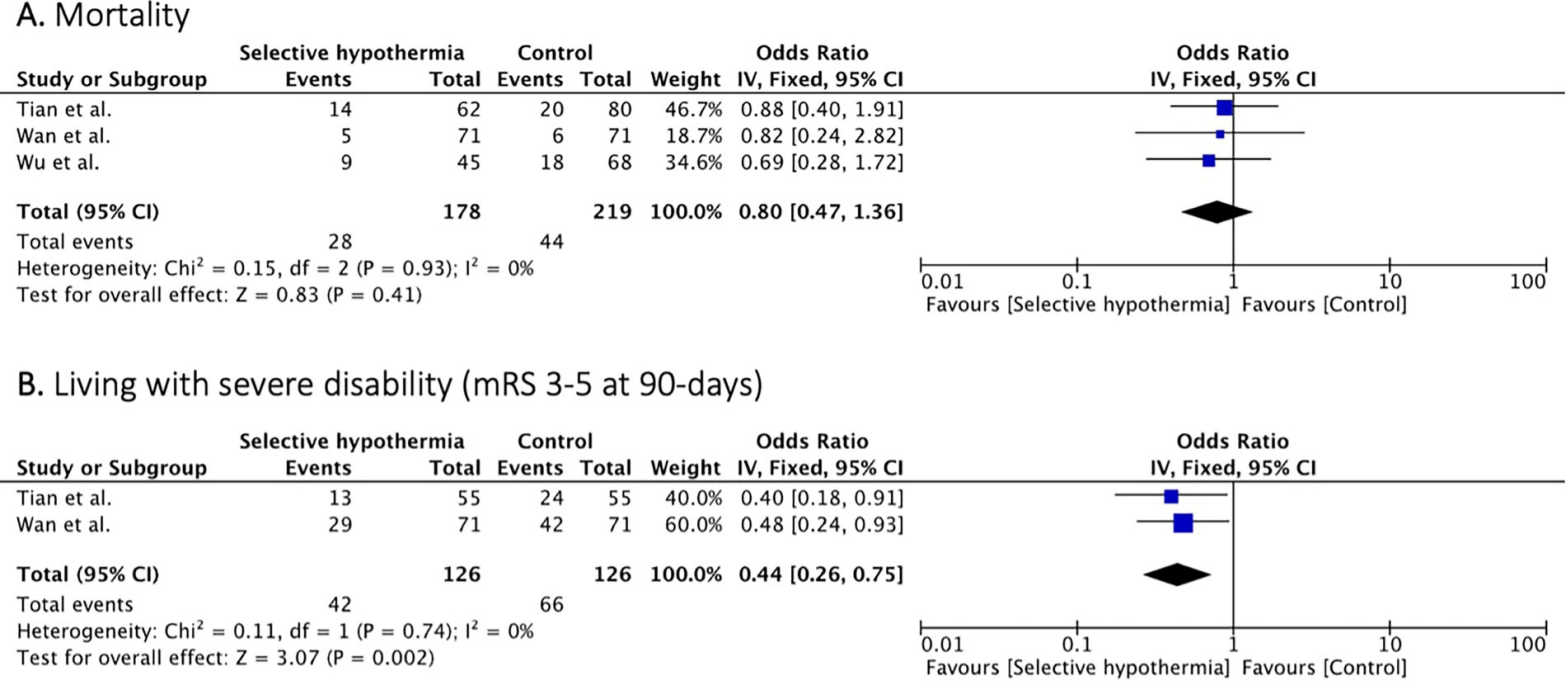
Forest plot of rates of mortality and severe disability (mRS 3-5) at 90 days.

Mortality was reported in three studies^19,22,23^ (397 patients). Mortality rates were similar between the two arms (OR, 0.80 [95% CI, 0.47 to 1.36]; p = 0.41) with no significant between study heterogeneity (I^2 ^= 0%, p = 0.93) (Figure 3A).

Two studies^19,22^ (252 patients) reported rates of patients living with severe disability (mRS 3–5 at 90-days). Patients treated with selective intra-arterial hypothermia had lower rates of severe disability compared to patients treated with EVT alone (OR, 0.44 [95% CI, 0.26 to 0.75]; p = 0.002) with no significant study heterogeneity (I^2 ^= 0, p = 0.74) (Figure 3B).

Safety parameters including rates of sICH (OR 0.84, [95% CI 0.47 to 1.48]; p = 0.54), pneumonia (OR 1.09, [95% CI 0.71 to 1.68]; p = 0.69), coagulation abnormalities (OR 0.75 [95% CI, 0.24 to 2.34], p = 0.62), and arterial spasm (OR 1.65, 95% CI 0.36 to 7.47]; p = 0.52), and urinary tract infection (OR 0.89 [95% CI, 0.55 to 1.43], p = 0.62) were comparable between groups (Table 3).

**Table 3.** Safety parameters.

Outcome	No. of Participants	OR (95% CI)	Heterogeneity (I^2^, p for Cochrane Q)
sICH	397	0.84 (0.47, 1.48)	0%, 0.99
Pneumonia	437	1.09 (0.71, 1.68)	0%, 0.95
Coagulation abnormalities	397	0.75 (0.24, 2.34)	0%, 0.98
Hematocrit	284	3.52 (0.70, 17.76)	0%, 0.87
Arterial spasm	437	1.65 (0.36, 7.47)	0%, 0.70
Urinary tract infection	397	0.89 (0.55, 1.43)	0%, 0.75

sICH indicates symptomatic intracerebral hemorrhage; OR, odds ratio.

## Discussion

In this systematic review and meta-analysis, the use of selective intra-arterial hypothermia during EVT for LVO was found to be safe and feasible. The intervention group showed higher rates of good functional outcomes and lower rates of severe disability compared to the control group. Additionally, patients receiving selective intra-arterial hypothermia had lower final infarct volumes. Importantly, no increase in complications was observed with selective intra-arterial hypothermia, and safety outcomes were similar between groups.

Therapeutic hypothermia holds promise as a potential neuroprotective agent for patients with AIS. However, due to safety concerns, particularly the risk of pneumonia,^17,18^ systemic hypothermia is not routinely recommended for AIS patients.^28^ Instead, a selective approach to hypothermia may be more appropriate. Our analysis revealed comparable rates of post-stroke pneumonia. Furthermore, while whole-body cooling can potentially impair the coagulation process, this effect may not be as pronounced with selective brain cooling. Yet, we observed slightly higher hematocrit rates in the intervention arm, but this did not reach statistical significance. This could be explained by hemoconcentration as a physiological response to localized cooling. The decrease in temperature may have possibly caused fluid to shift from the warmer intravascular spaces into the cooler extravascular spaces leading to an increase in the volume of red blood cells relative to plasma.^29^ Another possible explanation is plasma volume reduction through cold diuresis. Although, the response is typically seen in the context of systemic hypothermia, it may also have a role in localized intra-arterial hypothermia.^30^ Additionally, there was underreporting of patients experiencing chills or shivering. However, Li et al. reported no such events in either group.^21^

Although current endovascular techniques achieve successful reperfusion rates exceeding 80%, reperfusion does not consistently lead to the full recovery. Reperfusion alone may not fully restore the anatomical structures and metabolic functions of ischemic tissue to their baseline states. Paradoxically, reperfusion can trigger destructive mechanisms, resulting in reperfusion injury that exacerbates the effects of ischemia leading to infarct progression, intracranial hemorrhage, and unfavorable outcomes.^31^ In this analysis, it was observed that despite the comparable recanalization rates (Table S4 and Figure S1), the hypothermia arm demonstrated higher rates of favorable prognosis. In addition to the benefits of reperfusion, adding hypothermia may help induce a state of ischemic tolerance. This state aids in reducing cellular demand for oxygen and energy, with anti-excitotoxic, anti-inflammatory, anti-edematous, and anti-apoptotic properties.^31–34^

Despite the potential increase in procedural time metrics and delayed reperfusion, the use of adjuvant selective hypothermia has shown a more favorable prognosis at 90 days compared to EVT alone. This improved outcome can be attributed, in part, to the lower final infarct volume in the intervention group, as no significant baseline differences were observed. Moreover, the favorable prognosis was accompanied by reduced rates of severe disability, indicating an opportunity for improved outcomes following EVT. However, it is important to note that most patients included in these syntheses were derived from only two single-center studies with the same working group, which may limit the generalizability of the results and highlight the need for further investigation.

Selective brain hypothermia has been explored using devices and strategies such as intranasal selective hypothermia, transvenous endovascular cooling, extraluminal vascular cooling, and epidural cerebral cooling. Ongoing trials are currently evaluating different selective modalities, such as the Reperfusion and Cooling in Cerebral Acute Ischemia II (ReCCLAIM II) trial (NCT03804060), which utilizes an intravenous cooling system, and the Hybernia post-mechanical thrombectomy (post-mtech) trial,^35^ which examines a selective intra-arterial system. Preliminary results from the ReCCLAIM II trial were presented at the 10th European Stroke Organization Conference and demonstrated no significant functional difference between the study groups but a suggestion of lower final infarct volumes in the hypothermia group. For the purpose of this review, we specifically focused on intra-arterial cooling methods combined with EVT for LVO. Further research is needed to determine the optimal intra-arterial hypothermia strategy, including the timing of intervention (pre- or post-EVT), the duration of cooling, and whether continuous or interrupted cooling yields the best outcomes.

This review has several limitations that should be acknowledged. Firstly, the inclusion of study-level data from five Chinese single-center studies restricts the generalizability of our findings due to potential ethnic variability, differences in vascular risk factors, and the possibility of selection and reporting biases. This may also raise concerns about potential patients overlap, however this was not the case as confirmed by the corresponding authors. Secondly, the small sample size (less than 500) limits the interpretability of our results and hinders our ability to detect meaningful differences. Further well-designed prospective studies and RCTs with larger sample sizes are needed to validate our findings. Thirdly, there was considerable variability in the EVT techniques and hypothermia strategies among the included studies, which may introduce heterogeneity. However, despite low heterogeneity in the synthesis, the limited number of studies and patients included in our analysis restricts the generalizability. Finally, none of the studies attempted to measure or estimate the brain temperature changes during cooling. Therefore, whether these methods succeeded in achieving any degree of brain cooling is uncertain.

## Conclusion

This systematic review and meta-analysis provide initial evidence that supports the safety and feasibility of selective intra-arterial hypothermia when combined with EVT for LVO. This approach shows promise for advancing research on neuroprotective strategies for ischemic stroke.

## Supplemental Material

sj-docx-1-ine-10.1177_15910199241285157 - Supplemental material for Selective intra-arterial hypothermia combined with endovascular thrombectomy for large vessel occlusion: A systematic review and meta-analysis Supplemental material, sj-docx-1-ine-10.1177_15910199241285157 for Selective intra-arterial hypothermia combined with endovascular thrombectomy for large vessel occlusion: A systematic review and meta-analysis by Fahad Alturki, Ahmed Alkhiri, Bander Alsulami, Fawaz F. Alotaibi, Aser F. Alamri, Bader AlRuhaymi, Elyas M. Bakhuraybah, Fahad S. Al-Ajlan, Adel Alhazzani and Mohammed A. Almekhlafi in Interventional Neuroradiology
