# Supplementary material for: Selective intra-arterial hypothermia combined with endovascular thrombectomy for large vessel occlusion: A systematic review and meta-analysis
Source: Interv Neuroradiol. 2024 Sep 19;32(4):1741–8. doi: 10.1177/15910199241285157 (PMC11559715; doi:10.1177/15910199241285157)
Supplement: Supplementary file 1 [file sj-docx-1-ine-10.1177_15910199241285157.docx]

**Supplemental material**

____

**Table S1.** Risk-of-Bias assessment using RoB2 tool for randomized studies.

**Table S2.** Risk-of-Bias assessment using NOS tool for non-randomized studies.

**Table S3.** Search Strategy.

**Table S4.** Detailed baseline characteristics of patients in included studies.

**Figure S1.** Forest plots for recanalization rates.

**Table S1.** Risk-of-Bias assessment using RoB2 tool for randomized studies.

| **Study ID** | **Randomization** | **Deviation from intended intervention** | **Missing outcome** | **Measurement of the outcome** | **Selection bias** | **Overall risk of bias** |
| --- | --- | --- | --- | --- | --- | --- |
| Wan, 2022 | Some concern | Low risk | Low risk | Low risk | Some concern | Some concern |

**Table S2.** Risk-of-Bias assessment using NOS tool for observational studies.

| **Study ID** | **Selection** | | | | **Comparability** | **Exposure/Outcome** | | | **Quality Score** |
| --- | --- | --- | --- | --- | --- | --- | --- | --- | --- |
|  | **Representativeness of the exposed cohort** | **Selection of the non exposed cohort** | **Ascertainment of exposure** | **Demonstration that outcome of interest was not present at start of study** | **Comparability of cohorts on the basis of the design or analysis** | **Assessment of outcome** | **Was follow-up long enough for outcomes to occur** | **Adequacy of follow up of cohorts** |  |
| **Wu, 2018** | ★ | ★ | ★ | ★ | **★★** | ★ | **★** | **★** | 9 |
| **Chen, 2016** | ★ | **-** | ★ | ★ | **-** | ★ | - | - | 4 |
| **Li, 2023** | ★ | ★ | ★ | ★ | **★★** | ★ | **-** | ★ | 8 |
| **Tian, 2023** | ★ | ★ | ★ | ★ | **★** | ★ | **★** | **★** | 8 |

**Table S3.** Detailed Search Strategy.

| **Database** | **Searches** | **Results** |
| --- | --- | --- |
| Embase | ('stroke'/exp OR 'stroke' OR 'ischemic stroke'/exp OR 'cerebral infarction' OR 'acute ischemic stroke' OR ‘large vessel occlusion’ OR ‘LVO’) AND ('endovascular'/exp OR 'endovascular' OR 'thrombectomy'/exp OR 'thrombectomy' OR 'evt') AND ('cooling' OR 'hypothermia’ OR 'cold saline') | 462 |
| Medline | Ovid MEDLINE(R) ALL <1946 to May 17, 2024>  1 stroke$.mp. 401104  2 large vessel occlusion$.mp. 4667  3 LVO$.mp. 4727  4 endovascular$.mp. 78339  5 thrombectomy$.mp. 20919  6 evt$.mp. 4755  7 cooling$.mp. 50523  8 hypothermia$.mp. 50717  9 cold saline$.mp. 618  10 1 or 2 or 3 404103  11 4 or 5 or 6 93834  12 7 or 8 or 9 96139  13 10 and 11 and 12 178 | 178 |
| Cochrane | EBM Reviews - Cochrane Central Register of Controlled Trials <April 2024>  1 stroke$.mp. 73173  2 large vessel occlusion$.mp. 891  3 LVO$.mp. 471  4 endovascular$.mp. 5238  5 thrombectomy$.mp. 2395  6 evt$.mp. 645  7 cooling$.mp. 3317  8 hypothermia$.mp. 4718  9 cold saline$.mp. 166  10 1 or 2 or 3 73346  11 4 or 5 or 6 6683  12 7 or 8 or 9 7332  13 10 and 11 and 12 66 | 66 |

**Table S4.** Detailed baseline characteristics of patients in included studies.

| **Study ID** | **Age, Mean (SD)** | | **Male, n (%)** | | **Baseline NIHSS, Median (range)** | | **Recanalization, n (%)** | | **Symptom onset to groin puncture, min** | | **Symptom onset to recanalization, min** | | |
| --- | --- | --- | --- | --- | --- | --- | --- | --- | --- | --- | --- | --- | --- |
|  | **I** | **C** | **I** | **C** | **I** | **C** | **I** | **C** | **I** | **C** | **I** | **C** |  |
| Wu et al., 2018 | 61.9 (9.7) | 62.2 (10.8) | 28 (62.2) | 45 (66.2) | 17 (13-21) | 16 (11-19) | 35 (83.3) | 54 (85.7) | 278 (218-305) | 266 (210-289) | 367 (274-423) | 348 (262-400) |  |
| Wan et al., 2023 | 73.4 (12.6) | 72.8 (13.6) | 44 (62.0) | 40 (56.3) | 15 (7)* | 16 (8)* | NR | NR | 258 (71)* | 279 (60)* | 348 (80)* | 361 (92)* |  |
| Chen et al., 2016 | 58 (9.56) | - | 15 (57.7) | - | 18 (12-22) | - | 26 (100) | - | 383 (195-432) | - | NR | - |  |
| Li et al., 2023 | 56.1 (10.78) | 57.0 (10.57) | 16 (80) | 16 (80) | 14.1 (6.01)* | 13.4 (4.35)* | 16 (80) | 17 (85) | 450 (244.4)* | 414 (230.4)* | 450 (244.2)* | 414 (230.4)* |  |
| Tian et al., 2023 | 64 (57–77)§ | 66 (61–76)§ | 41 (66.1) | 48 (60.0) | 17 (15-20) | 15 (12-17) | 44 (88.0) | 46 (83.6) | 266 (208-295) | 253 (213-289) | 366 (285-443) | 341 (271-429) |  |

* data decribed as mean (standard deviation)
§ data decribed as median (range)
NIHSS indicates National institutes of health stroke scale; NR, Not reported; I, intervention; C, control.

**Figure S1.** Forest plots for recanalization rates.
